# Extracellular vesicles in onco-nephrology

**DOI:** 10.1038/s12276-019-0213-7

**Published:** 2019-03-15

**Authors:** Chiara Gai, Margherita A. C. Pomatto, Cristina Grange, Maria Chiara Deregibus, Giovanni Camussi

**Affiliations:** 1 Stem Cell Laboratory, Department of Medical Sciences, University of Turin, Turin, Italy; 2 2i3T Scarl, University of Turin, Turin, Italy

**Keywords:** Kidney

## Abstract

Extracellular vesicles (EVs) are important mediators of intercellular communication in cancer and in normal tissues. EVs transfer biologically active molecules from the cell of origin to recipient cells. This review summarizes the studies on EVs derived from renal cell carcinoma and from a subpopulation of CD105-positive renal cancer stem cells. While EVs from renal cell carcinoma show mild biological activity, EVs from renal cancer stem cells enhance tumor angiogenesis and metastasis formation. The effect is probably due to the transfer of proangiogenic RNA cargo to endothelial cells, which acquire an activated angiogenic phenotype. In vivo, treatment with EVs favors the formation of a premetastatic niche in the lungs. Moreover, EVs derived from renal cancer stem cells modify gene expression in mesenchymal stromal cells, enhancing the expression of genes involved in matrix remodeling, cell migration, and tumor growth. Mesenchymal stromal cells preconditioned with tumor EVs and then coinjected in vivo with renal cancer cells support tumor growth and vessel formation. Finally, tumor EVs promote tumor immune escape by inhibiting the differentiation process of dendritic cells and the activation of T cells. Thus, tumor-derived EVs act on the microenvironment favoring tumor aggressiveness, may contribute to angiogenesis through both direct and indirect mechanisms and are involved in tumor immune escape.

## Kidney cancer: Packages that promote aggression

Membrane-bound packages called extracellular vesicles (EVs) released by kidney cancer stem cells can make tumors more aggressive, promote the onset of cancer at other sites, and help tumors escape the anti-cancer immune response. Giovanni Camussi and colleagues at the University of Turin, Italy, review understanding of EVs from kidney cancer cells. EVs from cancer stem cells are especially effective in promoting cancer, unlike those from mature cancer cells. This is partly due to their ability to promote the formation of new blood vessels to sustain tumor growth. Some of the vesicles’ effects are mediated by transferring small molecules of ribonucleic acid (RNA) into other cells. These RNAs can regulate the activity of specific genes, promoting cancer. Studying patients’ EVs may assist cancer diagnosis and help predict the likely progression of the disease.

## Introduction

Cancer cells, as well as all other cells, are capable of releasing extracellular vesicles (EVs) into the extracellular space. EVs are vesicles surrounded by a lipid bilayer containing protein and nucleic acid cargo. EVs are shed in physiological and pathological circumstances. After release, EVs can reach close or distant sites by entering the circulation and can be found in all biofluids. The term “extracellular vesicles”, suggested by the International Society of Extracellular Vesicles (ISEV), designates a mixed population of vesicles with overlapping dimensions released by cells and typically distinguished into exosomes and microvesicles, ectosomes and shed vesicles based on their biogenesis^1^. Exosomes come from the membrane invagination of multivesicular bodies (MVBs); exosomes are vesicles of 30–150 nm in diameter secreted into the extracellular space after fusion of MVBs with the plasma membrane. The formation of exosomes partially relies on the endosomal sorting complex required for transport (ESCRT) complex^2,3^, but it may also take place independently from ESCRT, with the participation of tetraspanins in protein sorting^4^ or of ceramide^5^. The RAB proteins are other players involved in exosome biogenesis^4,6^. At variance, microvesicles are 100–1000 nm in diameter and directly bud from the plasma membrane. Vesicles shed from the plasma membrane may include vesicles released by normal cells, such as stem cells, which are in the nano-range (100–200 nm in diameter), and larger preapoptotic vesicles, which are released by injured cells. Apoptotic bodies are vesicles of 1000–5000 nm in diameter secreted by cells undergoing programmed death and containing nuclear fragments^7^.

In recent years, EVs have been profusely studied, and their roles in cell-to-cell communication, as well as their involvement in cell microenvironment homeostasis, have been recognized. In fact, EVs can exchange specific bioactive molecules, such as proteins and nucleic acids, among cells, influencing the phenotype and functions of recipient cells^7^.

EVs show different proportions of membrane lipid molecules, such as cholesterol, sphingomyelin, and ceramide, with respect to the cell of origin^6^ and carry various proteins involved in EV biogenesis. For example, EVs carry proteins involved in the formation of MVBs, such as TSG101, ALIX^3,8^ and clathrin, and proteins contributing to membrane transport and fusion, such as flotillins, annexins, and GTPases^6^. RAB proteins, involved in docking and fusion of EVs with recipient cells, and heat shock proteins, such as HSP90 and HSP70, are also present in EVs^4,6,7^. Of interest, tumor EVs convey mediators of oncogenesis, such as growth factors, oncoproteins, and immunomodulatory molecules, that may affect the tumor microenvironment and metastatic niche^9–11^.

The tumorigenic activity of EVs relies on their luminal cargo and on the assortment of transmembrane proteins involved in EV tropism, such as integrins interacting with the extracellular matrix. CD63, CD9, and CD81 tetraspanins are the most frequently mentioned exosome markers, but not all exosomes express these proteins; in addition, these tetraspanins may also be present in microvesicles and apoptotic bodies^12^. In addition to proteins, EVs may contain fragments of DNA of genomic and mitochondrial origin, single or double-stranded, carried on the surface or inside the EVs^13–16^. Moreover, they contain numerous classes of RNA, such as mRNA, microRNA, long noncoding RNA, mitochondrial RNA, transfer RNA, and ribosomal RNA^6,17–19^.

### Cancer-derived EVs

Evidence demonstrates that cancer cells release higher amounts of EVs with functional alterations compared to normal cells, probably due to biogenesis and cargo sorting deregulation. Different mechanisms possibly involved in increased EV production have been described, including the overexpression of syntenin^8,20^, RAB proteins^10^, ESCRT components^21,22^, and heparinase^23^. In addition, EV production can also be induced by a hypoxic microenvironment^24^, as well as by the activation of oncogenic signaling pathways, such as EGFRvIII^25^, h-RAS^26^, and proto-oncogene SRC^27^. The altered secretion and function of tumor EVs critically affects the cross-talk between cancer and surrounding tissues, engendering a favorable microenvironment for tumor development and invasiveness. In particular, tumor EVs influence normal and cancer cell behavior, promoting tumor growth through the stimulation of angiogenesis, the migration and invasion of cells, the development of premetastatic niche, the reduction of cell-to-cell adhesion, and the modulation of an immune response^11,28–30^ (Table 1).

**Table 1:** Role of cancer-derived EVs in tumor progression

Biological effect	Mechanism	Tumor	Refences
Stimulation of angiogenesis	Transfer of proangiogenic mRNAs/miRNAs	Renal carcinoma	11
	Transfer of miR-23a and upregulation of HIF-1α	Lung cancer	31
	VEGF upregulation	Renal carcinoma	32
	Induction of c-Kit, the receptor tyrosine kinase Tie2 and Met in bone marrow progenitors	Melanoma	10
	Transfer of sphingomyelin, MMPs and plasminogen activator	Fibrosarcoma	30
	Activation of SRC signaling	Chronic myeloid leukemia	33
	Transfer of EDIL-3 and activation of epidermal growth factor receptor signaling	Bladder cancer	35
Decrease in cell-to-cell adhesion	Reduction of E-cadherin and β-catenin expression	Bladder cancer	36
	Downregulation of tight junction protein ZO-1 mediated by miR-23a and miR-105	Lung and breast cancer	8, 31
Increase in cell migration/invasion	Transfer of KIT	Gastrointestinal stromal tumor (GIST)	41
	Transfer of mRNAs/miRNAs	Renal carcinoma	11
Development of premetastatic niche	Recruitment and reprograming of bone marrow progenitors, inducing the transforming growth factor β secretion and upregulating fibronectin production in surrounding hepatic cells Mediation of cancer stem cells stimulation of the premetastatic niche formation in the lungs	Pancreatic ductal adenocarcinomas (PDACs) Renal carcinoma	42 11
	Transfer of RNAs that activated Toll-like receptor 3, promoting neutrophil recruitment in the lungs	Lung cancer	43
	Induction of a prometastatic phenotype in bone marrow progenitors mediated by the expression of c-Kit, the receptor tyrosine kinase Tie2 and Met	Melanoma	10
Immune-modulation	Inhibition of dendritic cell and T-cell functions	Renal and nasopharyngeal carcinoma, pancreatic, lung and breast cancer	29, 44
	Promotion of tumor-supportive inflammation through the stimulation of cytokine secretion by macrophages	Gastric, breast, and prostate cancer	44

Indeed, tumor EVs have been reported to promote angiogenesis via modulation of different pathways. They shuttle proangiogenic mRNAs and microRNAs implicated in tumor progression and metastasis^11^. A recent study by Hsu et al.^31^ demonstrated that EVs released by cancer cells under hypoxic conditions overexpressed miR-23a, which induced enhanced angiogenesis by leading to an accumulation of HIF-1α in endothelial cells. In another study, tumor EVs were able to upregulate VEGF expression in endothelial cells, possibly via the downregulation of the hepatocyte cell adhesion molecule hepaCAM^32^. In melanoma, tumor EVs were shown to reprogram bone marrow progenitors toward a provasculogenic and prometastatic phenotype, inducing the expression of c-Kit, Tie2 and Met^10^. In chronic myeloid leukemia, instead, cancer EVs stimulated tube formation in vitro and in vivo through SRC signaling activation^33^. In renal cell carcinoma, EVs were enriched with azurocidin protein, which is involved in vascular permeabilization. Thus, tumor EVs were shown to modify the endothelial cell phenotype, disrupting vascular morphology^34^. In addition, EVs released by bladder-cancer patients were shown to be enriched with EDIL-3, which activated epidermal growth factor receptor signaling in cancer and endothelial cells, promoting their angiogenesis and migration^35^. In addition to their proangiogenic activity, EVs released by cancer cells have been recognized as important mediators of the epithelial-to-mesenchymal transition, favoring target-cell migration and invasion. Indeed, tumor EVs can target adherent junction molecules in tumor cells and reduce the expression of E-cadherin and β-catenin epithelial markers^36–38^. Moreover, vesicular miR-23a and miR-105 were shown to inhibit the tight junction protein ZO-1 (TJP1), thereby increasing vascular permeability and cancer migration of lung and metastatic breast cancers^31,39^. Indeed, by analyzing breast cancer cells, Harris et al.^40^ demonstrated that cells with higher metastatic potentials released EVs characterized by a unique protein signature and by an enhanced promigration activity in comparison to normal EVs^40^. In gastrointestinal stromal tumors, tumor EVs contained the oncogenic protein tyrosine kinase KIT and were shown to promote the invasion of the interstitial stroma, inducing a tumor phenotype in progenitor smooth muscle cells^41^. The increased migration capacity of cancer cells leads to metastasis, and tumor EVs were reported to be critically involved in premetastatic niche formation. In the liver, EVs released by pancreatic ductal adenocarcinoma cells were able to promote the recruitment of bone marrow-derived macrophages, inducing the transforming growth factor β secretion by Kupffer cells and the upregulation of fibronectin production by hepatic stellate cells^42^. For the lung metastasis niche, tumor EVs contained RNAs that activated Toll-like receptor 3 in lung epithelial cells, inducing chemokine secretion and promoting neutrophil recruitment in the lungs^43^. Finally, tumor-derived EVs mediate the promotion of tumor-supportive inflammation, stimulating the secretion of cytokines from macrophages^44^. Another tumor-promoting function that has been associated with EVs is the chemo-resistance. In fact, Qu et al.^45^ showed that EVs transport sunitinib outside the cell. They discovered that the EV-associated lncRNA competitively binds miR-34/miR-449 and promotes AXL and MET expression in RCC cells. Thus, they proposed EV-associated lncRNA as a biomarker and therapeutic target for sunitinib resistance.

### Renal cancer stem cells

Kidney malignancies represent the ninth most common cancer in men and the 14th most common cancer in women worldwide. More than 90% of kidney cancers can be classified as renal cell carcinoma (RCC), which arises from renal tubular epithelial cells and includes clear cell (70%), papillary (10–15%), and chromophobe (5%) carcinoma histologic subtypes. RCC is characterized by poor prognosis due to a high metastasis rate and resistance to both radiotherapy and chemotherapy^46^. Treatment limitations are mainly represented by the incomplete eradication of tumor cells due to cellular heterogeneity. In particular, the presence of a small subpopulation of cancer cells with stem cell features, called cancer stem cells (CSCs), is raising interest in the field as the major cause of tumor recurrence and resistance to therapy^47,48^. These cells can differentiate into all tumor cell types and drive tumor development, tumor growth and metastasis formation^47^. CSCs have been isolated from several cancers, including RCC^47–51^. Renal CSCs (rCSCs) are capable of self-renewal and contribute to tumor vasculogenesis, metastasis development and resistance to therapy^52^. To date, CSCs have been isolated from several renal tumors using different isolation techniques. Despite experimental heterogeneity, all renal CSC populations share important stem cell features and characteristics, such as tumor-initiating ability, which helps to define rCSC identity. In 2008, Bussolati et al. identified rCSCs as a cell population of less than 10% of the tumor mass positive for the mesenchymal marker CD105^49^. These cells also express other mesenchymal stem cell markers, such as CD73, CD90, CD44, CD29, CD146, and vimentin; embryonic renal marker Pax2; embryonic stem cells markers, such as OCT4, NANOG, Nestin, and Musashi; but lack differentiative epithelial markers, such as cytokeratin and the adult renal progenitor markers CD133^49^. The authors hypothesized that the rCSCs might derive from resident renal stem cells with mesenchymal characteristics or from an embryonic dedifferentiated progenitor cell^52^. In fact, rCSCs display several features, including clonogenicity, sphere generation and tumor-initiating ability. Moreover, rCSCs generate serially transplantable tumors and differentiate toward several lineages, such as epithelial and endothelial cells. A very low number of rCSCs implanted in vivo in SCID mice were able to recapitulate the tumor of origin. Of interest, several endothelial cells present within the tumor expressed the HLA class 1 antigen, suggesting that rCSCs not only originate from the epithelial compartment of the tumor but also contribute to the intratumor vessel structures^49^. Previous studies have shown that in normal kidneys there are cells with multipotent properties expressing the CD133 stem cell marker. Bruno et al.^53^ investigated whether the CD133-positive population isolated from cancer had tumor-initiating ability and whether it could be defined as a CSC population. The results showed that these cells were able to enhance tumor engraftment and growth when implanted together with renal cancer cells, but they were not tumorigenic *per se*. Similarly, Galleggiante et al.^54^ characterized RCC cells that stained positive for CD133, CD24, and copper transport protein 2, a membrane marker important in RCC cisplatin-based resistance and that lacked mesenchymal markers. These cells displayed self-maintenance and differentiating capabilities in vitro and promoted angiogenesis in vivo. In another study, CXCR4 + cells derived from a highly tumorigenic RCC cell line expressed embryonic stem cell makers (NANOG, SOX2 and OCT3/4), displayed sphere-forming ability, increased resistance to tyrosine kinase inhibitors and caused tumor growth in vivo^55^. Moreover, rCSCs have been isolated using functional approaches. Zhong et al.^56^ selected rCSCs from a renal cancer cell line based on their ability to grow in suspension and to form spheres. These cells were CD105-positive and expressed several stem cell genes (OCT4, β-catenin, BMI, and NANOG). Moreover, these cells had self-renewal ability and were resistant to irradiation and chemotherapeutic agents. Cells able to form spheres contained a CD44 + /CD24- subpopulation with enhanced aldehyde dehydrogenase activity. These cells showed CSC features, such as the expression of stem cell survival signaling pathways, mesenchymal and prometastatic genes, and resistance to radiation^57^. Addla et al.^58^, instead, identified rCSCs from human renal tissues using a dye-exclusion assay to select cells that could rapidly extrude dyes due to specific membrane transporters typically associated with stemness. These cells displayed a high proliferative potential and were able to produce differentiated spheroids.

### rCSC-EV characterization

After the discovery of rCSCs, Grange and coworkers^11^ isolated and characterized EVs from CD105-positive rCSCs (rCSC-EVs) and compared them to EVs from non-stem, CD105-negative renal cancer cells. To identify any differences, the two EV types were analyzed by zeta-potential and size and by electron microscopy. These two EV types showed similar size (10–100 nm) and zeta potential (22.4 ± 3.5 mV). On the other hand, FACS analysis revealed that both kinds of EVs expressed CD44 and adhesion molecules (α5- and α6-integrins), which were characteristic of the cells of origin, but only rCSC-EVs expressed CD105. Both EV types did not express HLA class I or CD73. The authors analyzed the RNA content of EVs by bioanalyzer, showing that both EVs carried RNA of different sizes, mostly small RNAs (below 80 nucleotides in length) but also longer RNAs. The ribosomal subunits 28 S and 18 S were nearly absent, as this usually occur in EVs^59–61^. Then, the miRNA content of EVs was screened by qRT-PCR, profiling 365 human miRNAs. Both EVs carried approximately 80–90 miRNAs, but with a statistically significant difference; the rCSC-EVs contained 24 upregulated and 33 downregulated miRNAs compared to EVs derived from a non-stem renal cancer cell population. The upregulation of several miRNAs carried by rCSC-EVs (miR-200c, miR-92, and miR-141) has been detected in other cancers, such as ovarian^62,63^, colorectal^64^, and prostate cancers^65^. Moreover, miR-29a, miR-650, and miR-151 detected in rCSC-EVs have been previously associated with tumor invasion and metastasis^66–68^. A previous comparison of renal carcinomas with normal renal tissue showed the upregulation of miR-19b, miR-29c, and miR-151 which are enriched in rCSC-EVs^69^. To better understand the role of miRNAs carried by rCSC-EVs, the authors performed gene ontology (GO) enrichment analysis on the 24 miRNAs upregulated in rCSC-EVs. They observed that these miRNAs were strongly related to biological processes, such as metabolic processes, transcription, cell adhesion molecules, and regulation of cell proliferation.

Moreover, rCSC-EVs, but not those derived from non-stem renal cancer cells, were shown to carry several mRNAs of proangiogenic genes, such as VEGF, fibroblast growth factor 2 (FGF2), angiopoietin 1, ephrin A3, MMP2, and MMP9^11^.

### rCSC-EV proangiogenic role

Following the observation that rCSC-EVs carry proangiogenic miRNAs, Grange et al.^11^ decided to investigate whether rCSC-EVs exert a proangiogenic effect. Both rCSC-EVs and EVs derived from non-stem renal cancer cell populations were equally incorporated by endothelial cells; however, only rCSC-EVs affected angiogenetic processes. In fact, rCSC-EVs significantly enhanced the formation of capillary-like structures in Matrigel, promoted cell invasion through Matrigel-coated transwells and promoted apoptosis resistance after doxorubicin treatment, whereas EVs derived from non-stem renal cancer cells were ineffective. Moreover, endothelial cells pretreated with rCSC-EVs favored the adhesion of renal tumor cells to the monolayer of endothelial cells. Interestingly, EVs derived from unsorted tumor cells showed a modest biological effect, which was greater than that of EVs derived from renal cancer cells deprived of the stem cell population or vehicle alone. This result was probably due to the presence of rCSCs in the total population derived from the primary tumor. Moreover, endothelial cells prestimulated with rCSC-EVs were embedded in Matrigel and injected subcutaneously in SCID mice. The pretreatment with EVs significantly increased the formation of capillary structures expressing human markers von Willebrand Factor and HLA class I, which are connected with the murine vasculature. Furthermore, repeated rCSC-EV injections for 5 days followed by the administration of renal tumor cells significantly enhanced the incidence of lung metastasis, compared to EVs derived from non-stem renal cancer cells, vehicle or RNase-treated rCSC-EVs. An enhanced expression of VEGFR1 protein and VEGF and MMP2 genes in lung endothelial cells, and MMP9 gene in lung tissue was observed after treatment with rCSC-EVs, but not with EVs derived from non-stem renal cancer cells.

Overall, these results indicate that EVs released specifically by rCSCs, and not by all tumor populations, were able to promote tumor angiogenesis and invasion. These vesicles may coordinate angiogenesis within the tumor microenvironment and promote tumor growth. In addition, they condition lung tissues, creating a premetastatic niche and a favorable environment for cancer cell adhesion. Finally, the RNA cargo analysis of rCSC-EVs and EVs derived from non-stem renal cancer cells support different biological effects of EVs. In fact, rCSC-EVs were enriched in protumorigenic miRNAs and proangiogenic mRNAs^11^.

### rCSC-EVs protumorigenic role and cross-talk with MSC

The biological effect of rCSC-EVs was further investigated by Lindoso and colleagues^28^ to study the possible cross-talk between rCSCs and MSCs. In fact, some studies reported that MSCs are recruited within the tumor and promoted tumor growth and angiogenesis^70–73^; however the mechanism is still unclear. Thus, the authors evaluated whether rCSC-EVs could promote MSC recruitment. MSCs preconditioned with rCSC-EVs showed significantly increased migration toward CSC-conditioned medium. It was observed that MMP1, MMP2, MMP3, COL4A3, CXCR4, and CXCR7 were significantly upregulated compared with unstimulated control MSCs after 2 weeks of stimulation. CXCR4 is involved in MSC migration^74,75^, and CXCR7 is involved in survival and paracrine actions of MSCs^76,77^, angiogenesis, modulation of the immune system and tumor invasion^78,79^^,^. MMPs are known to modulate matrix remodeling and are increased in many human cancers, where they modulate invasion, metastasis, growth, and angiogenesis^80^. Moreover, the COL4A3 gene can regulate cell adhesion, migration and metastasis in various tumors^81–83^. Indeed, the upregulation was maintained for another 2 weeks in MSCs cultured after the removal of EVs from conditioned medium, confirming the persistence of phenotypic changes observed in stimulated MSCs. Moreover, MSCs stimulated for 2 weeks with rCSC-EVs induced angiogenesis in endothelial cells and migration in renal carcinoma cell when plated on the opposite side of a transwell. By analyzing cytokines secreted by MSCs stimulated or not with rCSC-EVs, Lindoso et al.^28^ observed that the secretion pattern was different. A significant increase in IL-8, myeloperoxidase (MPO) and osteopontin (OPN) gene transcription was observed by real-time PCR, and the corresponding protein release was determined by ELISA. MPO participates in oxidative stress response in tumors^84^ and may support tumor development. IL-8 can mimic VEGF enhancing endothelial cells proliferation and survival^85^ and is associated with several signaling pathways involved in tumor cell proliferation^86^. OPN is known to mediate the cross-talk between MSCs and cancer cells and to promote MSC migration^87^. This molecule is highly expressed in the tumor stroma and is involved in signaling regulation processes linked to angiogenesis, metastasis and tumor growth in different tumors and in apoptosis resistance in RCC^88^. Furthermore, the authors studied the behavior of rCSC-EV-stimulated MSCs in vivo and their effects on tumor growth. Tumors coinjected with stimulated MSCs showed an increased size and weight, a higher number of vessels and a proliferative rate compared to tumors alone or tumors coinjected with unstimulated MSCs. In conclusion, Lindoso et al. provided new insights into the putative involvement of rCSC-EVs in tumor communication with stromal surrounding cells, such as MSCs. Moreover, rCSC-EVs induce epigenetic reprogramming of MSCs and favor tumor vascularization and growth.

### Role of rCSC-EVs in immune-modulation

Another important mechanism promoting tumor development is tumor immune escape. The tumor microenvironment inhibits the maturation and activation of dendritic cell (DCs), limits their role as antigen-presenting cells and reduces their ability to activate naïve T lymphocytes^89^. Grange et al.^29^ analyzed the ability of rCSC-EVs to modulate the behavior and differentiation of monocyte-derived DCs. At first, the authors evaluated the ability of rCSC-EVs and EVs derived from non-stem renal cancer cells to inhibit DC differentiation by culturing monocytes in the presence of EVs. rCSC-EVs interfered with the phenotype of monocyte-derived cells, with a reduced expression of activation markers CD83 and CD40, costimulatory molecules CD80 and CD86, antigen-presenting molecule HLA-DR, and adhesion molecules involved in T-cell contact. DCs that differentiated in the presence of rCSC-EVs had a significantly reduced ability to stimulate CD3 + lymphocyte proliferation and released a significant amount of IL-10 compared with control DCs. Moreover, the soluble nonclassical human leukocyte antigen G (sHLA-G) was significantly increased in the supernatant of monocyte-derived cells cocultured with rCSC-EVs. HLA-G is known to suppress the function of natural killer (NK) cells, T cells, and DCs^90^ and is associated with cancer immune escape^91,92^. Moreover, 50% of clear cell RCCs (ccRCCs) have shown HLA-G upregulation and the presence of sHLA-G in patients’ plasma^93,94^. Western blot analysis demonstrated the presence of sHLA-G within rCSC-EVs at a higher level compared to EVs derived from renal cancer cells deprived of the stem cell population. The addition of the sHLA-G blocking antibody to monocyte-derived cells incubated with rCSC-EVs induced a partial reversion of the inhibitory effect and increased the expression of CD86, HLA-DR, CD1a, and α5 integrin on monocyte-derived cells.

In conclusion, the results demonstrated that rCSC-EVs impair the maturation of DCs and inhibit the T-cell immune response, partially through EV-associated sHLA-G.

## Conclusions

**Fig. 1:**
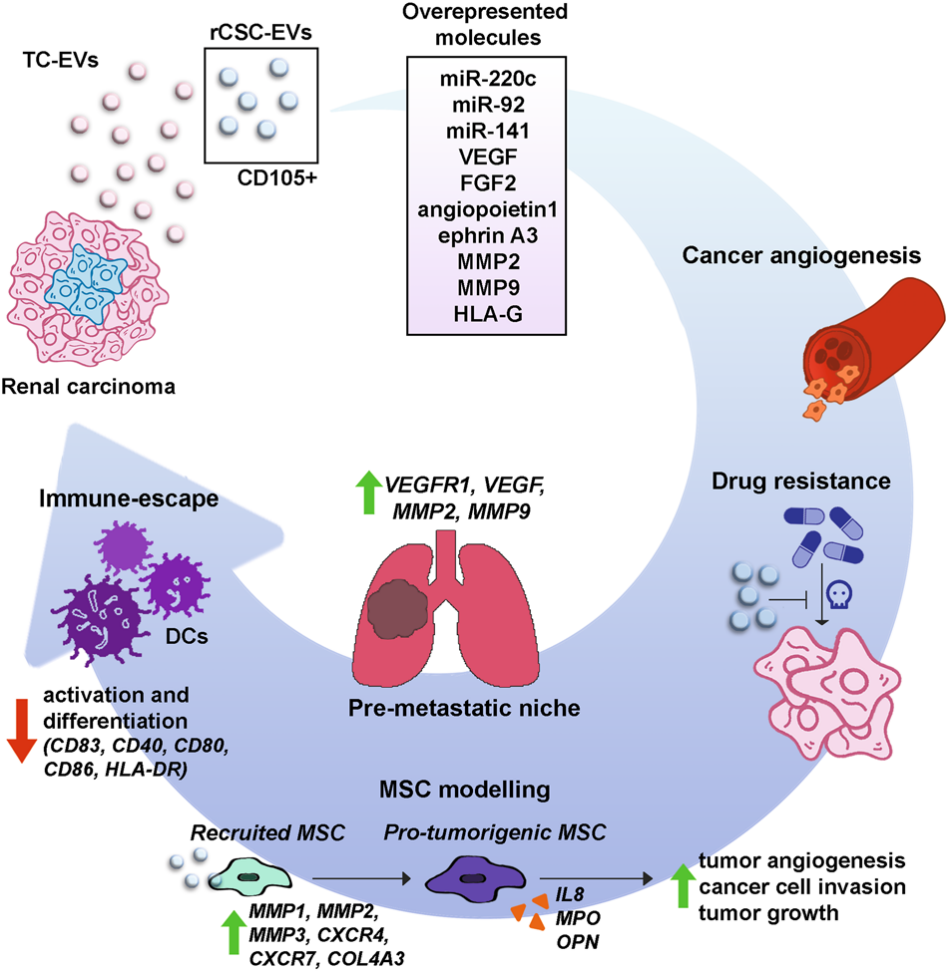
Role of rCSC-EVs in RCC. Renal carcinoma contains tumoral cells (TC) that are CD105-negative and cancer stem cells (rCSCs) that are CD105-positive. Renal cancer stem cells that are CD105-positive release EVs (rCSC-EVs) that are able to promote tumor growth. There are more than 24 upregulated miRNAs, including miR-200c miR-92 and miR-141, in CSC-EVs compared to tumoral cell-derived EVs (TC-EVs). Unlike TC-EVs, CSC-EVs carry several mRNAs of proangiogenic genes, such as VEGF, fibroblast growth factor 2 (FGF2), angiopoietin 1, ephrin A3, MMP2 and MMP9. Additionally, HLA-G protein was enriched in rCSC-EVs compared to that in TC-EVs. Moreover, rCSC-EVs support cancer development by several mechanisms. These vesicles were shown to promote tumor angiogenesis in vitro and in vivo and to promote apoptosis resistance following treatment with anticancer drug doxorubicin. Moreover, rCSC-EVs were proven to favor lung premetastatic niche formation through the upregulation of VEGFR1, VEGF, MMP2 and MMP9 in target tissue. In addition, rCSC-EVs can promote MSC migration inducing the upregulation of genes, such as MMP1, MMP3, CXCR4, MMP2, COL4A3 and CXCR7. MSCs stimulated with rCSC-EVs released IL-8, myeloperoxidase (MPO) and osteopontin (OPN) at higher concentrations, promoted cancer angiogenesis and tumor cell migration and increased tumor development in vivo. Finally, rCSC-EVs were shown to mediate cancer immunosuppression by reducing dendritic cell (DC) differentiation and activation. In particular, rCSC-EVs contained higher levels of HLA-G compared to TC-EVs and decreased the expression of activation markers CD83 and CD40, costimulatory molecules CD80 and CD86, and the antigen-presenting molecule HLA-DR in DCs

Taken together, these studies show that EVs released by rCSCs but not those derived from the more differentiated tumor cell population may influence the tumor microenvironment by acting on interstitial and endothelial cells and favor the formation of premetastatic niches. Moreover, rCSC-EVs may induce a protumorigenic phenotype in primed MSCs, and, by affecting the DC maturation and function of T cells, rCSC-EVs may favor tumor immune escape (Fig. 1). This is an outstanding example of how cancer stem cell-derived EVs are active players in the cancer microenvironment and can promote tumor growth and metastasis formation. Due to the emerging role of cancer EVs in tumor biology, several researchers are working to collect EVs from patients’ biofluids to identify new diagnostic, or even prognostic, biomarkers. As an example, De Palma et al.^95^ isolated urinary EVs, screened the RNA cargo and identified three mRNAs (GSTA1, CEBPA, and PCBD1) that were significantly reduced in ccRCC patients. This mRNA signature may be used for diagnostic screening. Moreover, the association between miRNAs and cancer was confirmed by the restoration of a gene expression level comparable to that of healthy controls after cancer removal. Two studies analyzed the expression of some miRNAs in sera EVs of RCC patients and identified miR-224^96^, miR-210, miR-1233, and miR-15a^97^ as being overexpressed in serum EVs; these miRNAs may eventually be suitable as diagnostic markers for the disease. This line of research is particularly important because the identification of sensible and specific serum or urinary EV biomarkers would be useful for the development of reliable, noninvasive and less expensive screening techniques for RCC.
